# State of Lunacy in England
*The Sixth Annual Report of the Commissioners in Lunacy to the Lord Chancellor. Pursuant to Act 8 and 9 Vic., cap. 100, sect. 88. Ordered by the House of Commons to be printed, 8th August, 1851.


**Published:** 1852-07-01

**Authors:** 


### Art. V.—STATE OF LUNACY IN ENGLAND.*

The Annual Reports of the Commissioners in Lunacy are always looked forward to with interest by all persons engaged in this department of our profession, inasmuch as they lay before us an official and authentic account of the existing state of lunacy and lunatic asylums throughout

* The Sixth Annual Report of the Commissioners in Lunacy to the Lord Chancellor. Pursuant to Act 8 and 9 Vic., cap. 100, sect. 88. Ordered by the House of Commons to be printed, 8th August, 1851.

England and Wales, and they record the history of their progressive improvement under the suggestions which the commissioners, from time to time, find it expedient to make. The sixth of these reports is now before us. It sets out with giving the statistical return of insane persons confined in asylums, hospitals, and licensed houses on the 1st of January, 1851, by which the aggregate number of private and pauper patients appears to have been—males, 7843; females, 8613; total lunatics, 16,456. The changes in the licensed houses in the metropolitan district were not, during the year 1850-51, very numerous, and require no special notice. The most important feature in the history of asylums during the year was the opening of two additional county asylums for the large and populous county of Lancashire—Rainhill and Prestwich asylums; the former capable of accommodating from 380 to 400 patients, the latter constructed for 450; but both these asylums may, with some slight alteration, be rendered capable of accommodating more, so that between Lancaster, Rainhill, and Prestwich, ample provision is made for many years to come for the lunatic poor of Lancashire. The result is, that the justices of the county will no longer grant any private house a licence for pauper patients, which we consider, for reasons we shall presently advert to, a very proper decision. The visiting commissioners report very favourably of the condition and arrangements made in these two new asylums—Rainhill and Prestwich. The medical superintendent of the former, we may observe in passing, is Mr. Thomas Eccleston, and of the latter Dr. Holland. In several counties—Lincoln, Bucks, Hants, Essex, &c., new asylums are in progress, and the commissioners recommend the transfer of all pauper patients, with as little delay as possible, from licensed houses to public establishments, and state that they have intimated to the proprietors of such private asylums their determination not to renew their licences for so large a number as they have hitherto done. The total number of private patients confined in asylums, hospitals, licensed houses, &c., on the 1st of January, 1851, was 4397, and the total number of paupers in asylums, registered hospitals, and licensed houses at the same date was 12,059. By the third annual report of the Poor Law Board we learn that the number of persons returned as lunatics, insane persons, and idiots, resident in the workhouses of 595 unions and of single parishes under boards of guardians in England and Wales, was, on the 1st of January, 1851, 5029. It is clear that as cases will increase with the increase of population, provision should be made as speedily as possible for the accommodation of these poor persons. It is indeed lamentable to think that so many are still domiciled in workhouses, where it is impossible they can receive proper medical and moral treatment. When the asylums referred to in this report as being in progress are completed, we are informed that the total number of pauper patients for whom accommodation will have been provided in county and borough asylums will be 13,929. This is so far, therefore, very satisfactory; indeed the history of lunatic asylums, as contained in the collective reports of the commissioners, sufficiently evinces continued progression. In the above estimate no account is taken of the Northampton Hospital—a voluntary institution, managed under the direction of the leading nobility, clergy, magistrates, and gentry of the county—which provides accommodation for nearly 200 paupers. On the 1st of January, 1851, this hospital contained 192 patients. The vigilance of the commissioners, and their determination to administer firmly and conscientiously the powers with which they have been invested in order to carry into effect the object contemplated by the legislature in passing the act 8 and 9 Vict., cap. 100, is sufficiently evinced by their exercising their power of insisting upon asylums being built in those cities and boroughs in which they are really required. Through their intervention, the authorities of the City of London have found themselves called upon to provide an asylum for the pauper lunatics chargeable to the several parishes within the jurisdiction of the City, and the matter is at this moment in the hands of a special committee. Among the different county and provincial asylums referred to in the report, the Manchester Royal Lunatic Asylum at Cheadle seems to demand some observation, inasmuch as the principle upon which it is founded clearly enough militates against the doctrine laid down by the commissioners themselves—viz., that private and pauper patients should be provided for in separate establishments—the one in licensed houses, the other in county asylums. Here, however, both private and pauper patients are domiciled under the same roof; and all medical men are well aware that the relations and friends of patients in the middle classes of society entertain a strong prejudice against sending them to an asylum where paupers are admitted. When this report was drawn up, this house at Cheadle, calculated to hold from 80 to 100 patients, contained only 22 private patients, and *one* pauper: total, 23. This asylum, it should be observed, is an offshoot, or branch of the Royal Infirmary at Manchester, and attended by all the inconvenience arising from its being managed by a committee connected with the infirmary, instead of the management being invested in one responsible party. Then, again, private patients are called upon to pay an entry fee, which, strange to say, is divided among the physicians of the infirmary non-resident in the asylum. This carries with it an air of traffic, to which we object. Then, again, a certain amount of payment in advance is required; and all these details are necessarily submitted to the committee, of which Mr. Harter is chairman. The commissioners in lunacy report favourably of the management of the Cheadle Hospital. With all deference, however, to the commissioners, we object *in limine* to the plan; for experience has abundantly proved that "remunerative patients" should not be constrained to live in a pauper institution, although the separation between the classes be quite perfect.

The commissioners in lunacy next advert to a somewhat curious legal disquisition which has taken place between them and the visiting justices in the county of Gloucester, who, with Mr. Purnell at their head, are indefatigable dabblers in the law of lunacy. We say this, bearing in mind their recent attack on the late Dr. Fox, of Northwood, near Bristol, and some other vexatious proceedings better consigned to the tomb of the Capulets. As this is a subject in which all proprietors and medical superintendents of lunatic asylums must feel interested, we subjoin the commissioners' own account of the decision that has been come to respecting the legal priority of orders of admission and medical certificates:—

"It will be satisfactory to your lordship," they observe, "to know that, in almost all our communications with the justices appointed to act as visitors of the various provincial establishments receiving lunatics, we have received the most ready and active co-operation.

"Some difference of opinion, however, has recently occurred between ourselves and the justices who are the visitors of a certain district in the county of Gloucester, relative to the interpretation of some portions of the existing statutes; the visiting justices holding that the order for the confinement of a lunatic should in every case be signed, and bear date prior to the medical certificates which accompany it. The ground of their opinion was, that in the form of the certificate the order and statement subjoined to it are referred to as 'the accompanying statement and order.'

"As this view of the subject was not only contrary to our own opinion (which had been communicated to the visiting justices of Gloucestershire), but was altogether at variance with the general practice throughout the kingdom, and as it was very desirable that the practice in this respect should be uniform, we submitted statements of the opinions of ourselves and the visiting justices to Secretary Sir George Grey, intimating our desire that he would cause the case to be laid before the law officers of the crown for their opinion. This was accordingly done, and the opinion of the law officers of the crown was found to concur with our own—namely, 'That so long as the provision of the 8 and 9 Vict., c. 100, is enforced, that no keeper of a private asylum shall receive a patient without the order and certificates required by that act, it is immaterial whether the order, in point of time, precede the certificates or the certificates the order.'

"Your lordship is aware that no priority, either as to order or certificates, is expressly required by any of the sections of the statutes now in force. Were any priority desirable, it would be that the medical certificates establishing the insanity of the patient should precede, in point of time, the order which directs his confinement; but it is manifestly for the public convenience that no such priority should be requisite, and it appeared to us that, looking at the words of the various enactments, and the general policy of the law on the subject, all that the legislature required was, that both the order and certificates should be obtained before the patient's admission, and that when complete they should together form one authority justifying the admission and detention of the patient.

"It may be remarked, that the 45th section of the act 8 and 9 Vict. c. 100, which prescribes the forms of the order and certificates (and which does not direct that either the order or the certificates should bear date prior to the other), is qualified in a very important particular by the 47th section of the same act, which enacts that a person may be received upon an order and *one* certificate only, provided such order state the special circumstances which *have prevented the person from being examined* by two medical practitioners; and that by the first section of the act 9 and 10 Vict. c. 84, it is enacted that it shall not be incumbent on a justice, &c., *to sign an order* for the confinement of a patient in all cases where the physician, &c. '*shall have signed the certificate* according to the form of the act,' and that every justice '*before* signing the order' shall satisfy himself as to the propriety of confining the lunatic, unless a certificate 'shall *have been* signed' by the medical officer of the lunatic's parish, *as well* as by the physician called in by the justice.

"Now, in order to carry out these two provisions, it is manifestly necessary that one or two certificates (as the case may be), should '*have been*' signed *previously* to the signature of the accompanying order and statement of particulars respectively, and it should be observed, that even in cases to which these provisions apply, the *same form of certificate* (containing the word 'accompanying'), is prescribed to be used by the acts of parliament."—*Report*, pp. 16, 17.

The commissioners in lunacy next briefly advert to the legal proceedings which, during the year, they felt it their duty to institute, and which, in each case, ended in convictions. One of them is of importance, as it involved the question of the admissibility of the evidence of a lunatic against an attendant, indicted at the Central Criminal Court for manslaughter. The case is that of *Re* Barnes, Peckham House. As we have given in a former number of this journal a full report of Mr. Collier's able argument in this case, with the decision of the judges, we do not consider it necessary again to enter into the question. The next point to which the commissioners in this report direct the attention of the legislature, is the establishment of a central asylum for the reception of criminal lunatics. In their former report, dated 30th June, 1850, they observed,—"the construction of lunatic asylums is so essentially different from that of prisons, that an effectual security against the escape of criminals cannot be provided without restricting the liberty of other patients, with whom they are necessarily associated, and materially interfering with that treatment and general arrangement which ought to be adopted for their benefit. Criminal patients have therefore escaped, and must continue to escape from asylums and houses licensed for the reception of the insane. As an instance of this we mention the fact, which was brought by us specially under the notice of secretary Sir George Grey, that a most active and cunning criminal patient escaped for the fifth time, from Hoxton House, in February last. Our objection applies especially to such lunatics as have been charged with the more heinous offences; and it has been frequently brought under our notice that the friends and relatives of patients, and also the patients themselves when conscious of their being associated with criminal lunatics, have considered such association as a great and unnecessary aggravation of their calamity." In their present report they again urge the early and special attention of the legislature to this subject. The last subject to which the commissioners in the present report address themselves, is the defective state of the law as affecting the property of lunatics. We have, they remark, "in former reports observed upon the very defective state of the law and its administration, as respects the property and income of lunatics, and the injustice and hardship thereby entailed upon them, their families, and others connected with, or having claims upon, them. Some strong remarks upon this subject are to be found in our 'further report,' (1847), page 28, and in our last (fifth) report, page 17, to which we earnestly solicit your lordship's attention. Frequent communications, with a view to the necessary legislation, have passed between the board and the home office, but we regret to say hitherto without any practical result. To the many persons who have appealed to us, from time to time, for advice and assistance towards the due protection and administration of the property and income of insane persons under certificates, we have only been able to express our regret that the provisions of the present law were inapplicable or inadequate to their professed object, and to hold out a hope that a legislative remedy would be shortly provided. We desire now again to press the subject upon your lordship, as one urgently calling for the earliest consideration and legislation, more especially as respects persons of small means, and also those whose mental malady is probably of a temporary character. It was with a special view to the benefit and protection of these classes of the insane, that the 95th and following three sections of the Act 8 and 9 Vict. c. 100 were framed; but unfortunately the intentions of the legislature have been in a great measure frustrated, partly in consequence of the limitation of the powers of the receiver, whom the Lord Chancellor is by the Act authorized to appoint, to those of the receiver of the estate of a lunatic found such by inquisition, but principally in consequence of the costs and expenses of the proceedings under the statute being so heavy, and so disproportionate to the limited amount of the property or income to be administered."

"Whilst we have had frequent occasion deeply to regret this state of things, for which, notwithstanding our representations, no remedy has been provided, *we have always refused our sanction to, and strongly discouraged, the transaction of any matters of business, or the execution of deeds or papers relating to property, by persons confined under certificates as insane. We have considered their position incompatible with the due exercise of their powers and rights as independent agents, although possibly they may, in some cases, be mentally capable of forming a correct judgment on the subject of their property, and disposing of the same reasonably and prudently.* In carrying out this principle we have felt and expressed our regret that the law, as now existing and administered, affords no effectual redress for the evils we have to deplore."

The commissioners in lunacy have, over and over again, called the attention of the Government to this most manifest defect in the present law of lunacy, and we are gratified to hear that a bill has been drawn up and submitted to Parliament to remedy the evil by the Earl of Shaftesbury; but we would go perhaps further than the commissioners in lunacy seem prepared to do. Under a commission of lunacy the committee of the person and estate is competent to protect the property of the lunatic in every respect; but, simply under certificate, we do not see why a reasoning lunatic—say a monomaniac, deranged on one point, with his senses clear and unimpaired upon others—should be deprived of any voice in the management of his affairs. We have seen that, by the decision of the judges, the evidence of a lunatic is admissible in a court of justice, where the life even of the accused may be at stake; why should not, under proper surveillance, the signature of a certified lunatic upon a subject he is capable of understanding, reasoning upon, and appreciating, be upon a legal document held valid? The delays created by the incompetency of such persons to sign documents relating to any matters of business, produce the greatest inconvenience and distress in many families; and if, as the commissioners themselves concede, any such persons are "mentally capable of forming a correct judgment on the subject of their property, and disposing of the same reasonably and prudently," we do not understand why they should be denied the privilege of doing so, whereby, in many minor transactions, the pecuniary resources of the patient would be at command, and enable the friends to provide additional comforts, without incurring the expense, as at present, of a commission in lunacy. The uncertainty which seems now to obscure the political atmosphere—the sudden change of administration, the early dissolution of the House of Commons, and consequent interruption for a few months to all legislation, may for awhile retard the progress of the bill introduced to the Upper House by the Earl of Shaftesbury; but every true philanthropist, and men of all shades of political opinion, will unite in throwing every possible protection round, not only the personal comfort, but the pecuniary interest and property of the afflicted lunatic, which ought to be rendered, at the least possible expense, available for his own advantage, as well as for the benefit of perhaps a dependent and bereaved family.

